# Genomic Instability and DNA Damage Repair Pathways Induced by Human Papillomaviruses

**DOI:** 10.3390/v13091821

**Published:** 2021-09-14

**Authors:** Takeyuki Kono, Laimonis Laimins

**Affiliations:** 1 Department of Microbiology-Immunology, Feinberg School of Medicine, Northwestern University, Chicago, IL 60611, USA; take.k1227@gmail.com; 2 Department of Otolaryngology Head and Neck Surgery, School of Medicine, Keio University, Tokyo 1608582, Japan

**Keywords:** HPV, oropharynx, DNA damage repair, APOBEC, mutations

## Abstract

Human papillomaviruses (HPV) are the causative agents of cervical and other anogenital cancers as well as those of the oropharynx. HPV proteins activate host DNA damage repair factors to promote their viral life cycle in stratified epithelia. Activation of both the ATR pathway and the ATM pathway are essential for viral replication and differentiation-dependent genome amplification. These pathways are also important for maintaining host genomic integrity and their dysregulation or mutation is often seen in human cancers. The APOBEC3 family of cytidine deaminases are innate immune factors that are increased in HPV positive cells leading to the accumulation of TpC mutations in cellular DNAs that contribute to malignant progression. The activation of DNA damage repair factors may corelate with expression of APOBEC3 in HPV positive cells. These pathways may actively drive tumor development implicating/suggesting DNA damage repair factors and APOBEC3 as possible therapeutic targets.

## 1. Introduction

Human papillomaviruses (HPV) are small, nonenveloped viruses that contain double-stranded DNA genomes of approximately 8 kb in length. More than 400 different genotypes of HPV have been identified, and each infects stratified epithelial tissues at distinct body locations [1]. Over one-third of HPV types infect cells in the genital tract and are sexually transmitted [2]. Twelve types of alpha genus including HPV 16, 18, 31, 33, 35, 39 and 45 among others are classified as high-risk type as they are associated with the development of anogenital cancers [3,4]. High-risk HPV (HR-HPV) infections are responsible for approximately 5% of all human cancers including almost all cervical along with other anogenital cancers, as well as over 60% of oropharyngeal cancers [5,6,7,8]. At the same time, over the last two decades in Western countries there has been a dramatic increase in the incidence of HPV induced oropharyngeal squamous cell carcinomas (OPSCC), and the number of cases will likely soon surpass those of cervical cancer [9,10]. Cervical cancers are caused by at least twelve high-risk types with HPV16 accounting for about 50%, HPV 18 by 25% while the remaining types are associated with less than 5% each [4]. In contrast, almost all HPV positive oropharyngeal cancers are the result of HPV16 [11]. HPV induced anogenital and oral cancers typically take extended periods to develop that can in some cases range up to several decades [12,13]. Since HPV vaccines only target initial infection but have no effect on existing lesions, a significant need exists to identify therapeutics to treat established HR-HPV infections.

## 2. Genome Organization

HR-HPV genomes encode an average of eight open reading frames (ORFs) that are expressed from polycistronic messages that are transcribed from one strand of viral DNA [14]. The individual ORFs in the viral genome are designated as early or late based on the stage of HPV life cycle in which they are initially expressed. The early gene products include E6, E7, E1, E2, E4 and E5. The E6 and E7 proteins are regulators of cell cycle progression in both undifferentiated and differentiated cells, and act as oncoproteins in the development of cancers. The E1 ORF encodes a virus-specific origin recognition protein that also has DNA helicase activity [15]. The E2 protein is a transcriptional regulator that binds to sites flanking the viral origin upstream of the start site of early viral transcripts and helps to recruit the E1 protein to the origin through complex formation [16]. The late gene products consist of the major capsid protein L1 and the minor capsid protein L2 which associate to form the virion shell [14].

## 3. The Differentiation-Dependent Life Cycle of HPVs

**Figure 1:**
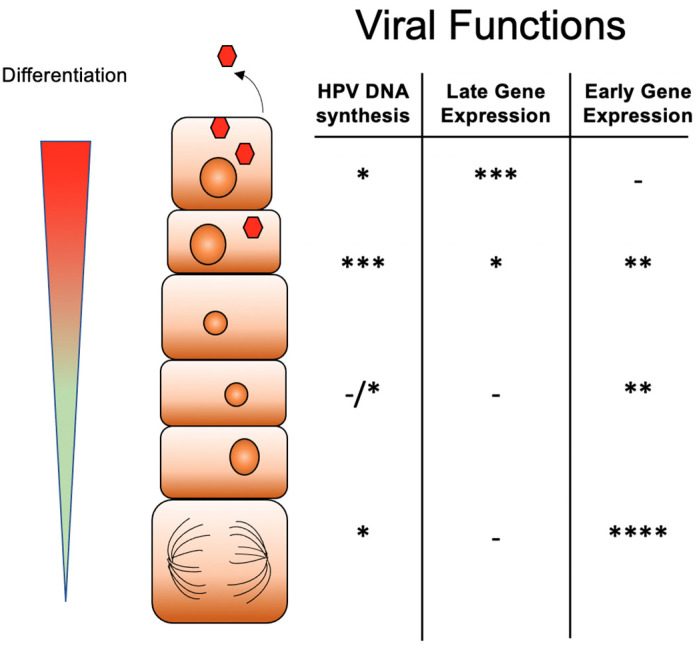
Differentiation-dependent life cycle of high-risk HPVs. Infection by HPV virions occurs into basal cells where genomes are established as multicopy episomes that replicate together with cellular replication. As HPV infected basal cells divide, one daughter cell migrates away from the basal epithelia to begin to differentiate. Upon differentiation late gene expression is activated while early transcription is reduced. The amplification of HPV genomes, virion assembly and release occur in suprabasal cells. The number of * indicates the level of activity with * indicating low level, ** medium, *** intermediate and **** high level.

The productive life cycle of HPV is closely linked to the differentiation state of the host epithelial cell and is regulated through the action of both viral as well as cellular proteins [14,17,18,19,20,21,22,23]. HPVs infect cells in the basal layers of stratified epithelia that become exposed following some form of microtrauma. Following entry, viral genomes are established in the nucleus as extrachromosomal elements or episomes at about 50–200 copies per cell [23,24,25,26] and HPV genomes are replicated in synchrony with cellular chromosomes in the S phase. HR-HPV positive basal cells can remain persistently infected until they are cleared by the immune response or progress to cancers. In HPV infected epithelia, a subset of differentiating suprabasal cells remain active in the cell cycle and progress through the S phase into G2/M where productive replication of HPV genomes or amplification to thousands of copies occurs in G2/M (Figure 1) [27]. As will be discussed below, viral genome amplification is dependent upon activation of the ataxia telangiectasia mutated-dependent (ATM) and the ataxia telangiectasia and Rad3-related (ATR) DNA repair pathways. The L1 and L2 capsid proteins are expressed from the late promoter concurrent with amplification resulting in virus assembly and release of virions from the uppermost layers [28].

## 4. Host DNA Damage Repair Pathways

**Figure 2:**
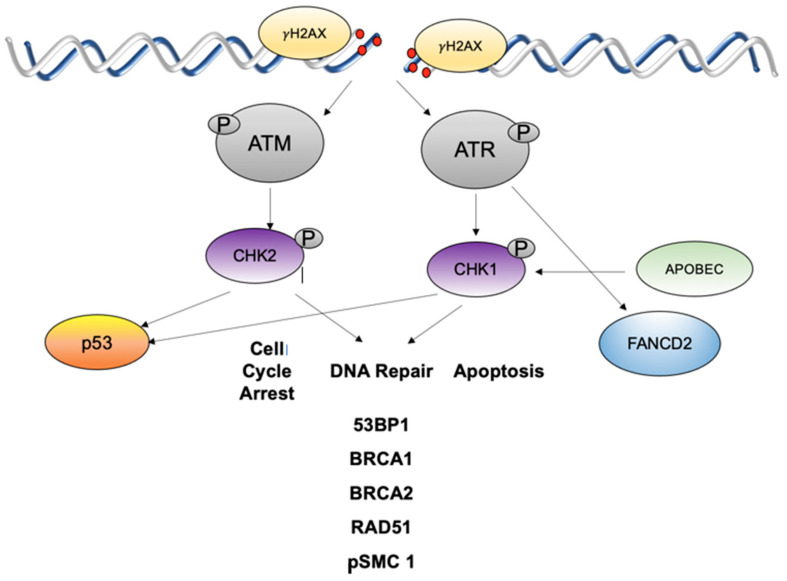
ATM and ATR DNA damage pathways are activated in HPV infections in response to high levels of DNA breaks. ATM is autoactivated which leads to phosphorylation of CHK2 that then activates a series of downstream effectors to mediate cell cycle arrest and DNA repair or apoptosis. ATR is activated by single strand breaks or stalled replication forks and acts to phosphorylate CHK1 which then activates downstream targets including FANCD2. γH2AX is a histone that is phosphorylated by ATM or ATR and flanks sites of DNA breaks.

The DNA damage response consists of a network of cellular signaling pathways that facilitate the repair of damaged cellular DNA to maintain genomic integrity. This includes mechanisms to repair damaged DNA and, in some cases, induce apoptosis or senescence, as well as activation of an innate immune response [29]. The two main ways to mediate repair are through the non-homologous end joining pathway and the homologous recombination repair pathway. The latter includes the ATM and ATR pathways. Both ATM and ATR proteins are serin/threonine protein kinases from the phosphatidylinositol 3-kinase-related kinase (PIKK) super family. The ATM pathway is activated in response to DNA double-strand breaks (DSB) that are initially recognized by the MRN (MRE11-RAD50-NBS1) complex [30]. The MRN complex recruits ATM to sites of double strand breaks leading to its activation by autophosphorylation and acetylation by Tip60. Activated ATM then induces phosphorylation of downstream proteins including CHK2 and the histone H2A variant γH2AX [30]. pCHK2 and pATM also activate additional repair factors such as BRCA1, BRCA2 and SMC1 among others (Figure 2) [29]. These factors repair breaks through the homologous recombination (HR) pathway in which genetic material is copied using templates from sister chromatids. Activation of the ATM pathway also leads to phosphorylation of p53, the tumor suppressor and cell cycle regulator protein, resulting in cell cycle arrest or apoptosis [31].

The ATR pathway is activated in response to replication stress and the presence of single-stranded DNA (ssDNA) at stalled replication forks. ssDNA is bound by the replication protein A (RPA), which recruits ATR and its binding partner, ATRIP, to sites of damage [30]. The recruitment of TopBP1 facilitates phosphorylation of ATR, and leads to activation of downstream effector proteins including CHK1 kinase along with components of the Fanconi Anemia (FA) pathway as well as BRCA1 and RAD51 [32]. The FA pathway is activated in response to DNA interstrand cross-links (ICL) that can form between bases on two opposite DNA strands. This type of lesion can impede the progression of DNA replication forks leading to stalling and collapse. The stalled fork at the ICL is recognized by the FANCM-FAAP24-MHF complex. The FA core complex then ubiquitinates the FANCI-FANCD2 complex, which primes ubiquitinated FANCD2 (FANCD2-Ub). FANCD2-Ub localizes with DNA repair proteins, including γH2AX and BRCA1, at sites of damage and recruits proteins, such as FAN1 nuclease, BRCA2 and RAD51 to facilitate DNA repair [33,34]. ATM can also phosphorylate FANCD2, however, this induces an S-phase arrest [30,35]. While the ATM and ATR pathways are activated in response to different forms of DNA lesions, they target several common downstream effectors [36]. 

## 5. The DNA Damage Response and the HPV Life Cycle

Activation of the ATM and ATR DNA damage repair pathways is critical for the completion of the differentiation-dependent HPV life cycle [22,37,38]. Both ATM and ATR kinases are constitutively activated in cells that maintain complete HPV genomes as well as those that only express E6 and E7 [37,39]. This indicates that viral replication itself is not critical for activation of these pathways and that the action of viral proteins alone is sufficient. While both ATM and ATR are activated in both undifferentiated and differentiated cells, ATM is important only for viral amplification and has little effect on stable maintenance of episomes. In contrast, ATR activation is necessary for both stable maintenance as well as amplification indicating these pathways contribute in unique ways to viral replication [22,38]. Both pathways are constitutively activated by E6 and E7 as well as high-level expression of E1 [14]. It is, however, unclear if similar activation effects are seen when E1 is expressed at physiological low levels from complete viral episomes. Activation of these pathways results in the recruitment of downstream effectors such as pCHK2, γH2AX, BRCA1 and RAD51 to discrete nuclear foci that also contain HPV genomes [37,40,41]. Importantly these factors are bound to HPV genomes and are necessary for viral replication (Table 1). Another critical DDR factor is SMC1 which is phosphorylated by ATM and ATR in both undifferentiated and differentiated HPV positive cells. SMC1 forms complexes with the insulator DNA binding protein CTCF which is important for DNA looping and knockdown of either factor blocks HPV genome replication [42]. These complexes also bind the topoisomerase TOP2β which, as will be discussed below, is responsible for many of the DNA breaks in HPV positive cells [42].

In addition to its direct effects on DNA damage repair, ATR also indirectly regulates expression of a set of cellular genes. Activation of the ATR pathway in HPV positive cells is the result of complex formation of ATRIP and TOPBP1 which are all recruited to viral replication centers [43]. TOPBP1 is also phosphorylated by ATR and this is important for its parallel role as a transcriptional activator where it regulates genes such as E2F1 and p73 [44]. In HPV positive cells, ATR also induces phosphorylation of the autophagosome regulator p62 which leads to repression of the transcription factor GATA4. This results in downregulation of inflammatory gene expression as well as that of interferon kappa. The ATR/p62 signaling pathway is also critical for HPV viral replication and likely contributes to progression to cancer [45]. Increased levels of FANCD2 proteins are also present in the nuclei of HPV positive cells. In particular, FANCD2 proteins are preferentially recruited to viral genomes as compared to cellular sequences, and colocalize with a distinct population of DNA repair proteins including γH2AX and BRCA1, but infrequently with phosphorylated SMC1. Importantly, FANCD2 is essential for replication of HPV episomes [46].

The mechanisms responsible for constitutive activation of DDR pathways in HPV positive cells are beginning to be understood. Cells that stably maintain HPV episomes or those that express only E6 or E7 exhibit significantly enhanced levels of DNA breaks in comparison to normal cells [39,47]. In HPV positive cells, DNA breaks are present in both cellular and viral sequences but are rapidly repaired in HPV episomes due to the preferential recruitment of homologous DNA repair factors such as RAD51 and BRCA1. Interestingly, over half of the DNA breaks in HPV positive cells are due to the action of topoisomerases such as TOP2β [48]. TOP2β is a type II topoisomerase that binds to SMC1 and CTCF complexes at topologically associated domains (TADS) to relieve torsional stress arising during transcription or replication. TOP2β induces a transient double strand DNA break while remaining tethered to one end of DNA to allow passage of the other strand. This is followed by religation but failure to rapidly complete this process leads to double strand DNA breaks that are then repaired by DNA repair pathways. CTCF/SMC1/TOP2β binding sites are present in all HPVs and are largely located in the late regions or in the E2 ORFs [39,47,48]. The levels of TOP2β are substantially increased in HPV positive cells and it is responsible for inducing the majority of DNA breaks in HPV positive cells leading to substantial activation of DNA repair pathways. Knockdown of TOP2β blocks stable maintenance of episomes but has no effect on cellular replication, suggesting TOP2β is specifically important for viral replication [48]. Overall, HPV manipulates the DNA damage response to promote viral replication and the completion of its life cycle.

## 6. Progression to Cancer

Infections by high-risk HPVs in the genital tract produce asymptomatic lesions or dysplasias that are graded according to morphologic changes observed in the differentiation pattern. Cervical intraepithelial neoplasia (CIN)1 is characterized by koilocytic changes whereas CIN2 and CIN3 define moderate and severe dysplasia, respectively. Importantly, examination of biopsy materials revealed that the distribution and levels of DNA damage repair (DDR) factors increase with the grade of cervical lesion [49]. This suggests that activation of the DNA damage response in HPV positive genital epithelia may also be important during progression to cancer. Whether the DDR pathways are fully functional or have become impaired as lesions progress to cancers is still unclear. In contrast to the cervix, it has been hard to identify precancerous dysplasia HPV positive lesions in oropharyngeal epithelium, so it has not been possible to determine if similar increases in activated DDR pathways are seen. Large scale studies examining non-malignant tonsil samples have either failed to detect the presence of HPV genomes or found only low prevalence [50,51,52,53]. The most common site of HPV induced oropharyngeal squamous cell carcinoma (OPSCC) is the tonsillar reticular crypt epithelium and it may be not permissive for productive HPV infections but may allow for primarily transforming HPV infections [54]. The reason why transforming HPV infections are common in tonsils is unclear, but some insights may be deduced from our knowledge of HPV effects in cervical cancer.

The expression of both high-risk E6 and E7 is necessary for the immortalization of cells. The high-risk E7 proteins are localized to the nucleus where they bind the retinoblastoma, Rb, proteins and induce their rapid degradation. In normal cells, Rb proteins bind inhibit the action of E2F transcription factors that regulate expression of genes involved in replication in the S-phase. As cells transit into the S-phase, cyclin-kinase complexes phosphorylate Rb leading to release of E2F factors and activation of expression of replication genes. By inducing the degradation of Rb, E7 constitutively activates E2F, which leads to rapid entry into the S-phase [6]. This loss of Rb also leads to stabilization of the tumor suppressor, p53, that can result in cell cycle arrest and apoptosis. The high-risk E6 proteins have evolved to counteract the effects of these increased levels of p53 by recruiting it into a complex with the cellular ubiquitin ligase, E6Ap, leading to its rapid turnover. E6 also functions to activate expression of the catalytic subunit of telomerase, hTert, which is suppressed in normal cells but often activated in cancers [55]. The activation of hTert leads to maintenance of telomeres. The combined action of E6 and E7 in targeting p53, hTert and Rb is necessary for immortalization of cells infected with high-risk HPVs. The action of these two viral oncoproteins is, however, not sufficient to transform cells, which also requires the activation of cellular proto-oncogenes such as c-myc though mutation. One additional consequence of E7 mediated degradation of Rb is an increase in the levels of the cyclin dependent kinase inhibitor p16 which leads to a dependence on this factor for cell survival [56]. The increased expression of the p16 tumor suppressor is also an excellent biomarker for HPV-induced cancers.

HPV genomes are maintained as extrachromosomal episomes in precancerous lesions; however, they are frequently found integrated into host chromosomes in cancers [13]. Viral integration can occur with single or multiple copy genomes. Genome integration often occurs such that the E1 or E2 open reading frames have been disrupted, leading to enhanced expression of E6 and E7 as a result of loss of E2 mediated transcriptional repression [57,58]. Integration of the viral genome into the host’s DNA appears to be a crucial step for HPV mediated carcinogenesis [59,60]; however, not all HPV induced cancers have integrated genomes, indicating that other mechanisms such as additional somatic mutations are important. Since activation of the ATR pathway is necessary for the stable maintenance of episomes, it is possible that alterations in this or related pathways play a critical role in mediating integration. While studies have shown that increased levels of activated DNA damage repair factors are detected during progression, it is still not clear whether their activities are diminished.

## 7. Mutation Signatures in HPV Positive Cancers

Despite high level activation of DNA damage repair pathways, HPV positive lesions accumulate large numbers of mutations that contribute to progression to cancers. Somatic mutations have been recognized as a key mechanism of carcinogenesis by inducing driver mutations in a number of genes. Whole exome sequencing analyses of cervical cancer biopsy materials have identified recurrent somatic mutations in genes such as EP300 FBXW7, PIK3CA, TP53, MAPK, PTEN, ERBB2 and STK11 [61]. Interestingly, these mutational signatures are predominantly TpC to T/G mutations or CpG to T mutations. Of these, more than half of the non-silent mutations are TpC to T/G mutations [61]. In contrast, the most common genetic changes in HPV induced OPSCC are activating mutations and amplifications of the oncogene PIK3CA. Although PI3KCA is also often altered in HPV negative OPSCC [62], two specific C to T mutations in HPV positive lesions occur predominantly in hotspots at E542K and E545K that are implicated in PIK3CA kinase activation [63,64,65]. Less frequent alterations in HPV positive OPSCC include loss of chromosomal loci which contain the tumor necrosis factor receptor-associated factor 3 (*TRAF3*) and *ATM* genes [66]. In addition, amplification at 20q11, the location of the *E2F1* gene, is found in approximately 20% of HPV positive OPSCC while in only 2% of HPV negative OPSCC [63]. Although TP53 is frequently mutated in HPV negative OPSCC, this mutation is rarely seen in HPV positive OPSCC due to the reduced levels induced by E6/E6AP action. Similar to what is seen in cervical cancers, C to T mutations are prominent in HPV positive cancers in oropharynx, while the transversions associated with smoking, i.e., mutations that change purine nucleotide to another purine (A to G) or a pyrimidine nucleotide to another pyrimidine (C to T) at CpG sites are more frequent in HPV negative OPSCC [63].

## 8. APOBECs and HPV Positive Lesions

APOBECs are a family of cytidine deaminases that function to convert cytosine to uracil in ssDNA. At least 11 different types of deaminase are present in humans and contain either a single (APOBEC1, 3A, 3C, 3H and 4) or double (3B, 3DE, 3F and 3G) cytidine deaminase domain [67,68,69]. In normal cells, APOBEC proteins are expressed at low levels which increase upon viral infection through the activation of interferon signaling. APOBEC proteins were originally considered as innate immune anti-viral factors by inducing mutations into foreign RNA or DNA introduced with viral infections. In addition to endogenous retroviruses and retroelements, APOBECs also affect many viruses such as herpesviruses, parvoviruses hepatitis B and HPV. Analysis of data from TCGA revealed that HPV-positive head and neck as well as cervical cancers contain high levels of APOBEC3-mediated driver mutations [70,71]. Both APOBEC3A and 3B enzymes are upregulated in HPV-positive epithelial cells and target the same TpC dinucleotide motifs. The high frequency of mutations mediated by APOBEC3A and 3B in HPV positive cancers suggest they may contribute to malignant progression [72]. Furthermore, APOBEC3 deaminase activity is casually associated with helical domain hotspot mutations in PIK3CA gene, an oncogenic driver gene [73]. While APOBEC3 enzymes have the potential to mutate HPV genomes, clinical isolates rarely show evidence for hypermutation. Recent work has shown that APOBEC proteins have additional roles as mRNA editing factors as well as in the induction of DNA damage repair pathways.

**Figure 3:**
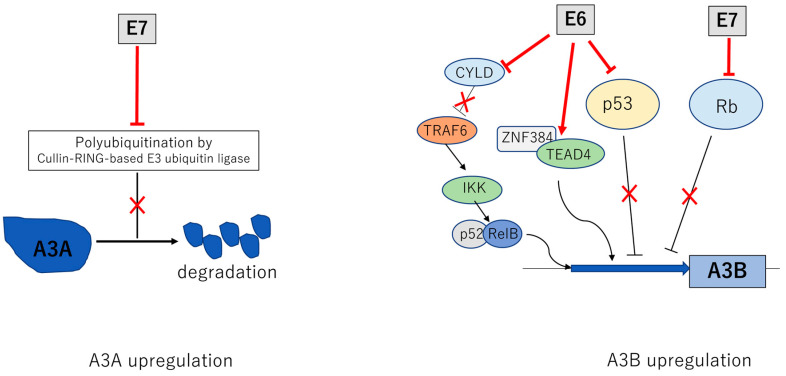
Activation of APOBEC3A (A3A) and APOBEC3B (A3B) by E6 and E7. Multiple mechanisms by which E6 and E7 increase levels of A3A and A3B. E6 and E7 act to increase transcription of A3B while E7 stabilizes A3A by blocking the action of a cullin E3 ubiquitin ligase. The inverted T indicates a direct inhibitory interaction with a viral protein leading to a blockage in the activity of the factor as indicated by an ✕

Persistent infection by HPV results in APOBEC induced mutations in cellular DNAs that can contribute to malignant progression [72]. HR-HPV E6 can upregulate APOBEC3B expression while low-risk E6 variants do not encode this ability [74]. E6 activates APOBEC3B expression by targeting the TEAD4 /ZNF384 complex that binds to the proximal promoter region [75,76]. APOBEC3B transcription also can be activated through E6 effects on NF-κB signaling [77]. Under hypoxic conditions that are commonly observed in cervical or head and neck cancer, E6 stimulates the ubiquitination and proteasomal degradation of the cylindromatosis (CYLD) deubiquitinase, a negative regulator of the NF-κB pathway that blocks TRAF-mediated IKK recruitment and activation leading to enhanced expression of APOBEC3B [78]. Furthermore, E6 can activate APOBEC3B in combination with E7 through degradation of p53 and Rb that have inhibitory activity on APOBEC3B promoter [79,80]. Overall, HR-E6 proteins have been shown to be potent regulators of APOBEC3B that act through several pathways (Figure 3). HPV positive cancers also exhibit increased levels of APOBEC3A which is the result of E7-mediated protein stabilization through inhibition of cullin-RING-based E3 ubiquitin ligase which mediates APOBEC3A degradation [81,82] (Figure 3). Kondo et al. found that integration of HPV genomes during malignant progression of oropharyngeal cancers is accompanied by increased APOBEC3A levels [83,84].

Several studies have suggested a linkage between DDR pathways and APOBECs. The repair of DNA breaks by the ATR pathway often generates ssDNAs which can be targeted by APOBECs [85,86,87]. Treatment of cancer cells with either ATM or ATR inhibitors leads to a reduction in the levels of APOBEC3 activation [88]. Furthermore, treatment with CHK1 inhibitors reduced the levels of APOPEC3B transcription [89] while increased expression of APBEC3A leads to enhanced levels of DNA breaks and γH2AX [90]. Activation of DDR pathways in HPV positive cells also corelates closely with increased levels of APOBEC3B [85]. In particular, components of ATR and FA pathways are increased HPV positive OPSCCC cells that also contain enhanced levels of APOBEC3B as compared to HPV negative cancers and normal cells. This suggests a possible linkage between APOBEC3B and DDR activation in HPV positive cells [85]. Although APOBEC3 proteins can function as antiviral factors, the high levels of APOBEC3 detected in cells that stably express HPV proteins suggest they may have a positive role in viral pathogenesis and is an area for future study.

Recent studies have suggested that DDR inhibitors such as ATR and Chk1 inhibitors may sensitize tumor cells with high levels of APOBEC to cell death [89,91,92]. Furthermore, APOBEC mutagenesis also induces high levels of neoantigen that can provoke an immune response in cancer cells [93]. In breast cancer models, genetic knockdown of APOBEC3B improved the durability of Tamoxifen treatment of ER+ xenograft tumors in mice [94]. Although identification of specific inhibitors against APOBEC3A and 3B are still ongoing, the development of APOBEC3A-ssDNA and APOBEC3B-ssDNA co-crystal structures may be helpful for developing structure-based molecule inhibitors that could be useful in cancer therapy [95,96].

## 9. Summary

High risk HPV proteins manipulate cellular pathways to promote their viral life cycles. HPVs constitutively activate the ATM and ATR DNA repair pathways and preferentially recruit these factors to viral genomes for productive viral amplification. Persistent infection by HPV results in increased levels of APOBEC3 proteins that result in TpC mutations in cellular DNAs that contribute to malignant progression. Targeting of APOBEC and/or DDR factors in HPV related cancers may be effective therapeutic targets.

## Figures and Tables

**Table 1:** List of DDR factors activated in HPV positive cells and their roles in binding of viral genomes, stable maintenance replication in undifferentiated cells and amplification upon differentiation. ND: not determined.

DDR Factor	Binds HPV Genome Replication	Stable Replication	Required for Amplification
pCHK2	Yes	No	Yes
pCHK1	ND	Yes	Yes
RAD51	Yes	ND	Yes
BRCA1	Yes	ND	Yes
FANCD2	Yes	Yes	Indirectly
γH2AX	Yes	ND	ND
pSMC1	Yes	ND	Yes
